# Decreased CD3+CD56+ Natural Killer T Lymphocytes and Increased Human Leukocyte Antigen-DR+ Cells in the Inflamed Area of Pouchitis in Ulcerative Colitis Patients

**DOI:** 10.7759/cureus.70066

**Published:** 2024-09-24

**Authors:** Masaya Iwamuro, Takehiro Tanaka, Masahiro Takahara, Toshihiro Inokuchi, Sakiko Hiraoka

**Affiliations:** 1 Department of Gastroenterology and Hepatology, Okayama University Graduate School of Medicine, Dentistry, and Pharmaceutical Sciences, Okayama, JPN; 2 Department of Pathology, Okayama University Hospital, Okayama, JPN

**Keywords:** flow cytometry, immunohistochemistry, lymphocytes, pouchitis, ulcerative colitis

## Abstract

Background: Pouchitis is an inflammatory condition that affects the ileal pouch during ileal pouch-anal anastomosis surgery. Despite its clinical significance, precise immunological mechanisms underlying pouchitis remain unclear. This study aimed to investigate the lymphocyte profile in the ileal pouch of patients with pouchitis compared to those with familial adenomatous polyposis (FAP) and ulcerative colitis without pouchitis using flow cytometry and immunohistochemical techniques.

Methods: We prospectively analyzed endoscopic biopsy specimens from the ileal pouches of 15 patients and categorized them into three groups: FAP, ulcerative colitis with an inflammation-free pouch (UC-I), and ulcerative colitis with ulcers and/or erosions in the pouch (UC-UE). Flow cytometry was used to assess various T-lymphocyte markers, including cluster of differentiation (CD) 4, CD8, CD56, and human leukocyte antigen (HLA)-DR. Immunohistochemistry was performed to visualize the spatial distribution of CD3+, CD56+, and HLA-DR+ cells in the pouch mucosa.

Results: We observed significantly reduced CD56+/CD3+ and CD8+/CD3+ ratios in the UC-UE group compared to those in the FAP group, indicating a disruption in natural killer T-cell populations. Immunohistochemical analysis revealed that the spatial distribution of lymphocytes differed among the non-inflamed mucosa, dense lymphocyte infiltration, and lymphoid follicles, with these components frequently intermingling. CD56 + cells were less abundant in areas with dense lymphocyte infiltration, whereas HLA-DR+ cells were more abundant.

Conclusion: Our study revealed a decrease in CD56+ natural killer T cells and an increase in HLA-DR+-activated T cells in areas with dense lymphocyte infiltration, suggesting an association between these cells and pouchitis in ulcerative colitis. The distinct patterns observed in non-inflamed mucosa, areas with dense lymphocyte infiltration, and lymphoid follicles underscore the need for further analyses of these three segments to elucidate the immunological mechanisms underlying pouchitis.

## Introduction

Pouchitis is an inflammation that occurs in the ileal pouch, a surgically constructed internal reservoir made from the ileum after the removal of the colon and rectum. This surgery, known as ileal pouch-anal anastomosis, is typically performed in patients with conditions such as ulcerative colitis or familial adenomatous polyposis (FAP) that necessitate colon removal. Pouchitis manifests with symptoms such as abdominal pain, increased stool frequency, urgency, and occasional bloody stools. While the precise etiology of pouchitis is not fully understood, potential factors include bacterial overgrowth or immune dysregulation within the pouch [1-5]. This study aimed to elucidate the distinctive characteristics of lymphocytes in pouchitis using flow cytometric analysis of T lymphocytes and immunostaining of the ileal pouch mucosa.

## Materials and methods

**Figure 1:**
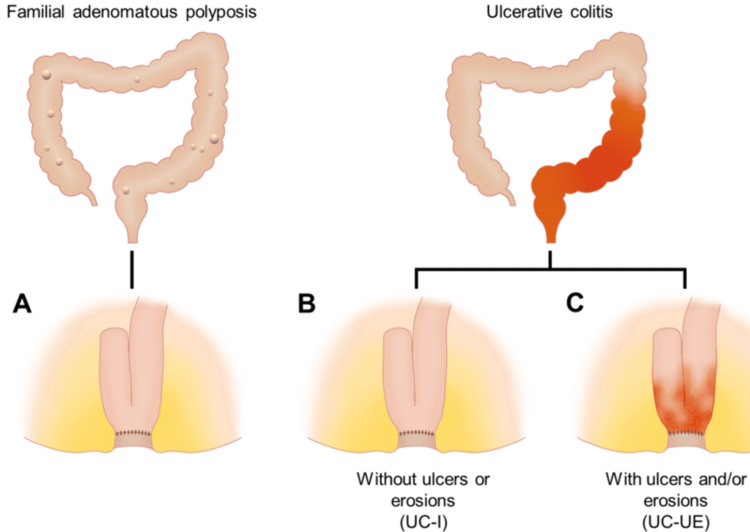
Diagram of the study patients. This figure illustrates the study groups used for the comparative analysis of lymphocyte characterization in the pouch mucosa. The three groups include (A) pouches in patients with familial adenomatous polyposis, (B) pouches in ulcerative colitis patients without ulcers or erosions, and (C) pouches in ulcerative colitis patients with ulcers and/or erosions. Image credits: Iwamuro M, Tanaka T, Takahara M, Inokuchi T, Hiraoka S.

Between March 2022 and June 2023, we prospectively performed flow cytometry on endoscopic biopsy specimens from 15 patients who had undergone ileal pouch construction. Written informed consent was obtained from all the participants. The inclusion criteria were as follows: (i) individuals who had undergone ileal pouch-anal anastomosis for FAP or ulcerative colitis, and (ii) the final stage of ileal pouch-anal anastomosis involving reconnecting the pouch to the anus and closing the ileostomy, was completed if a staged surgical approach was used. To characterize the lymphocytes in the pouch mucosa, comparative analyses were conducted among the three groups: (i) pouches in patients with FAP, (ii) pouches without ulcers or erosions in patients with ulcerative colitis, and (iii) pouches with ulcers and/or erosions in patients with ulcerative colitis (Figures 1A-1C). The presence or absence of ulcers and erosions was determined endoscopically.

For each of the 15 patients, two specimens were obtained using disposable biopsy forceps from the pouch mucosa during endoscopy. Lymphocytes were isolated from each biopsied specimen using a one-step lymphocyte isolation procedure, as reported in our earlier studies [6,7]. To profile isolated T lymphocytes in patients via flow cytometry, we analyzed the expression of various markers: cluster of differentiation (CD) 4 for helper T cells, CD8 for cytotoxic T cells, CD25 and CD127 for regulatory T (Treg) cells, CD56 (also referred to as neural cell adhesion molecule) for natural killer cells, CD7 for mature T cells, programmed death receptor-1 (PD1 or CD279) as a central inhibitory receptor, CD30 (tumor necrosis factor receptor superfamily member 8) for activated T cells, human leukocyte antigen DR isotype (HLA-DR), and C-C chemokine receptor type 4 (CCR4 or CD194) for type-2 helper T cells. Furthermore, we examined the expression of CD45RA and CD62L to classify lymphocytes into subpopulations, including effector memory T cells, central memory T cells, and naïve T cells.

Subsequently, to clarify the lymphocytes' localization, we performed hematoxylin and eosin staining and immunohistochemistry on another biopsy sample. The antibodies used were CD20 for B cells, CD3 for T cells, CD56, and HLA-DR. The images were converted to black-and-white monochromatic images for a clear visualization of the localization of CD3-, CD56-, and HLA-DR-positive cells. The conversion was performed using the Color Threshold function in the ImageJ software (version 1.54 g, National Institutes of Health, Bethesda, MD, USA).

Statistical analyses were performed using the JMP Pro (v.17.0.0; SAS Institute Inc., Cary, NC, USA). Student’s t-test or F-test was used to compare the two population means. For multiple comparisons, statistical analysis was performed using one-way analysis of variance, followed by the Tukey-Kramer post-hoc test. Statistical significance was set at p < 0.05. Numerical values are presented as means ± standard deviation. This study adhered to the principles of the Declaration of Helsinki and was approved by the Ethics Committee of Okayama University Hospital (2203-039). The study protocol was registered in the UMIN Clinical Trials Registry (UMIN000047219).

## Results

Patient characteristics

**Figure 2:**
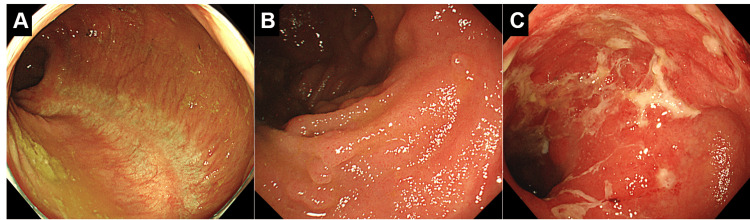
Representative endoscopic pictures of the ileal pouch. Ileal pouches in a patient with familial adenomatous polyposis (A), a patient with ulcerative colitis with an inflammation-free ileal pouch (B), and a patient with ulcerative colitis with ulcers and/or erosions in the ileal pouch (C).

The clinical characteristics of the enrolled patients are summarized in Table 1. The sex distribution was as follows: there were three women in the FAP group (Figure 2A); two men and one woman in the ulcerative colitis patients with inflammation-free ileal pouch (UC-I) group (Figure 2B); and six men and three women in the ulcerative colitis patients with ulcers and/or erosions in the ileal pouch (UC-UE) group (Figure 2C). No signs of inflammation were observed endoscopically in the ileal pouches of any patient in the FAP group. The median age was 51 years (range, 24-73 years) in the FAP group, 49 years (range, 45-50 years) in the UC-I group, and 39 years (range, 28-84 years) in the UC-UE group. Flow cytometry analysis was performed at a median of 9.9 years after ileal pouch construction in the FAP group, 10.5 years in the UC-I group, and 5.6 years in the UC-UE group.

**Table 1:** Patient characteristics. FAP, familial adenomatous polyposis; UC-I, ulcerative colitis with an inflammation-free ileal pouch; UC-UE, ulcerative colitis with ulcers and/or erosions in the ileal pouch.

	FAP	UC-I	UC-UE
Sex			
Male	0 (0%)	2 (67%)	6 (67%)
Female	3 (100%)	1 (33%)	3 (33%)
Age (median, years)(range)	51 (24–73)	49 (45–50)	39 (28–84)
Years after ileal pouch construction (median)	9.9	10.5	5.6

Lymphocyte composition in the ileal pouch

**Figure 3:**
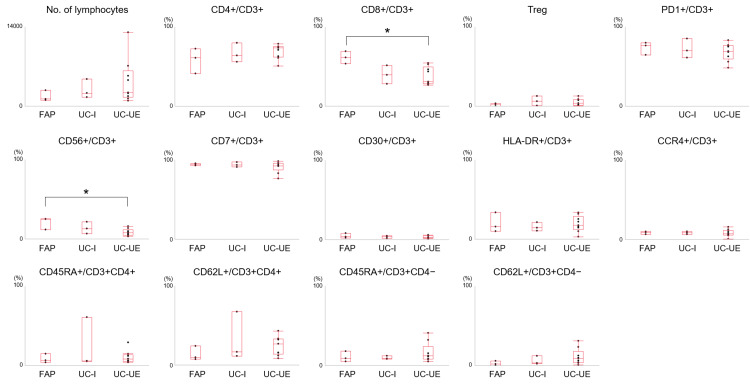
Flow cytometry analysis. Regulatory T (Treg) cells were defined as CD3^+^CD4^+^CD25^+^CD127^low/−^ cells. *, p < 0.05. FAP, familial adenomatous polyposis; UC-I, ulcerative colitis patients with inflammation-free ileal pouch; UC-UE, ulcerative colitis patients with ulcers and/or erosions in the ileal pouch.

The flow cytometry results for all samples are shown in Figure 3. Comparison between the FAP and UC-UE groups revealed that the CD8+/CD3+ ratio was decreased in the UC-UE group compared to the FAP groups (37.8 ± 3.6% vs. 61.4 ± 6.3%). The ratio of CD56+ to CD3+ cells was also lower in the UC-UE group compared to the FAP group (8.2 ± 1.9% vs. 20.7 ± 3.3%). No difference was observed in lymphocyte composition between the ileal pouches of the UC-I and UC-UE groups.

Immunostaining of the ileal pouch mucosa

Immunostaining was performed to determine the localization of lymphocytes within the ileal pouch mucosa. We used antibodies against the B cell marker CD20 and T cell marker CD3, in addition to CD56, which showed significant differences in our flow cytometry analysis (Figure 3). We also included HLA-DR, a marker of activated T cells, in the immunostaining analysis, as our previous studies revealed a significantly higher proportion of HLA-DR+ T lymphocytes in the intestinal mucosa than in the peripheral blood of patients with ulcerative colitis [8].

**Figure 4:**
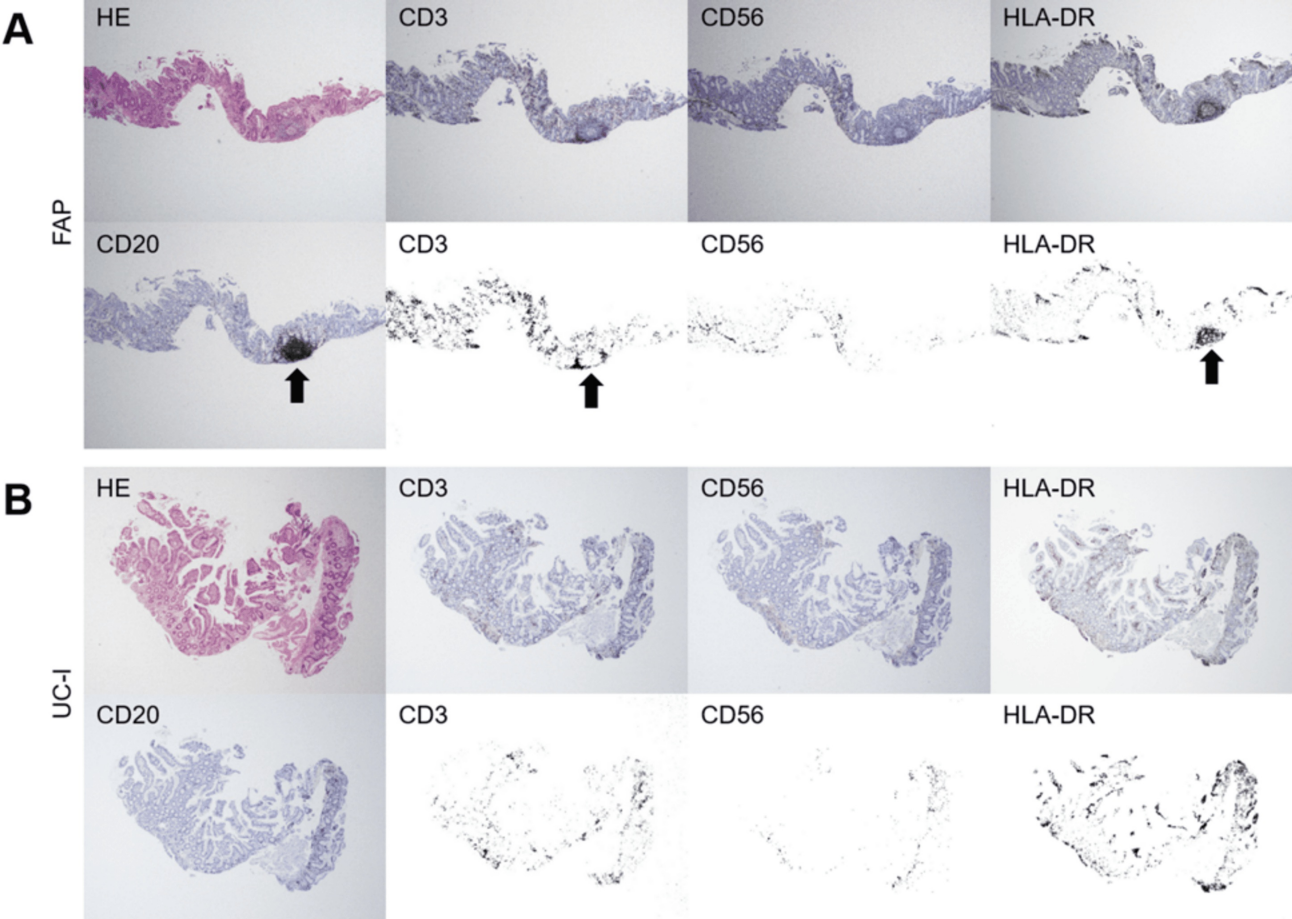
(A, B) Representative histological images of ileal pouch in a patient with familial adenomatous polyposis (FAP) and inflammation-free ileal pouch in a patient with ulcerative colitis (UC-I). Arrows indicate lymphoid follicles. Magnification: ×4.

**Figure 5:**
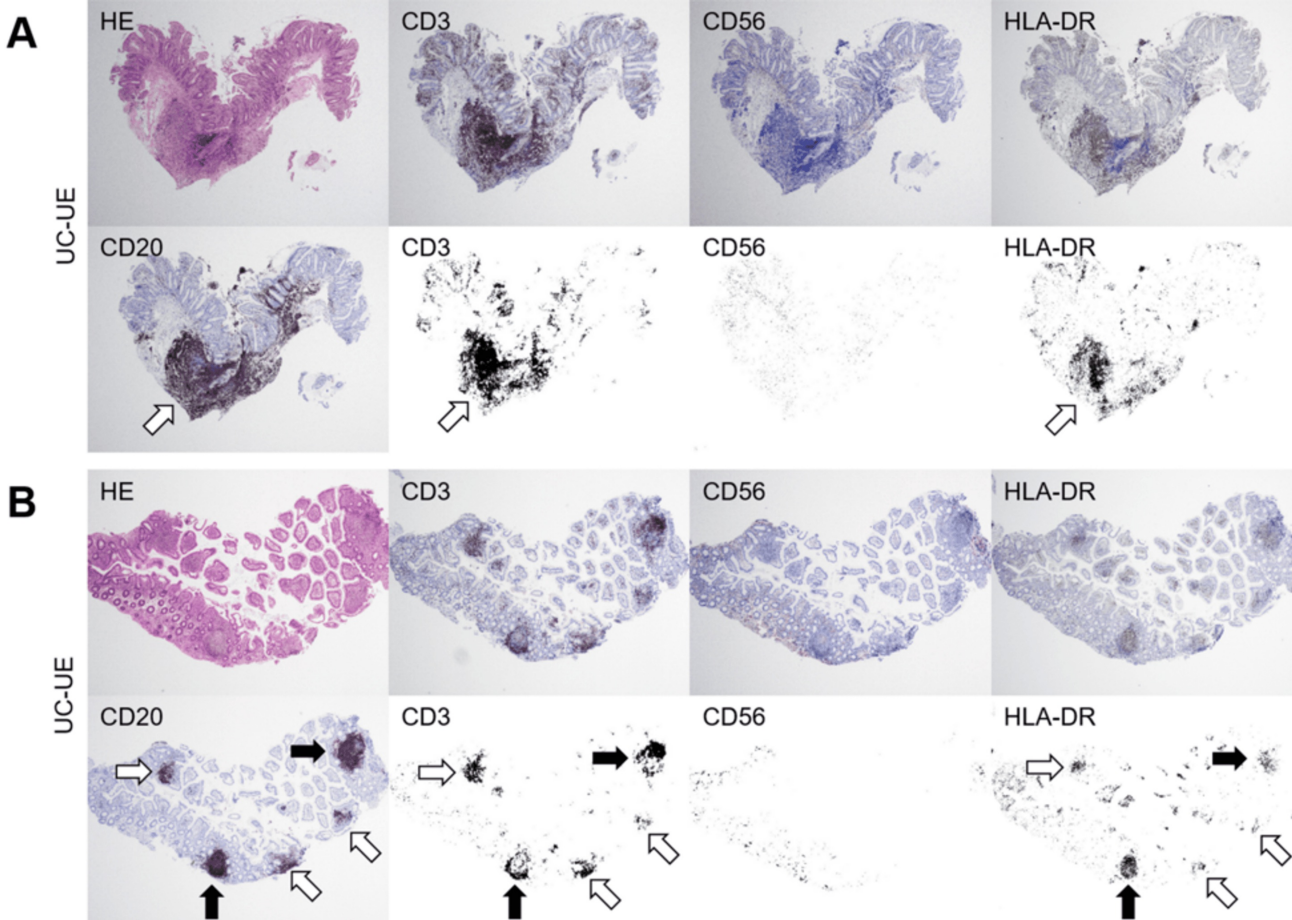
(A, B) Representative histological images of ileal pouches with ulcers and/or erosions in patients with ulcerative colitis. White arrows designate dense lymphocyte infiltration, while black arrows indicate lymphoid follicles. Magnification: ×4.

**Figure 6:**
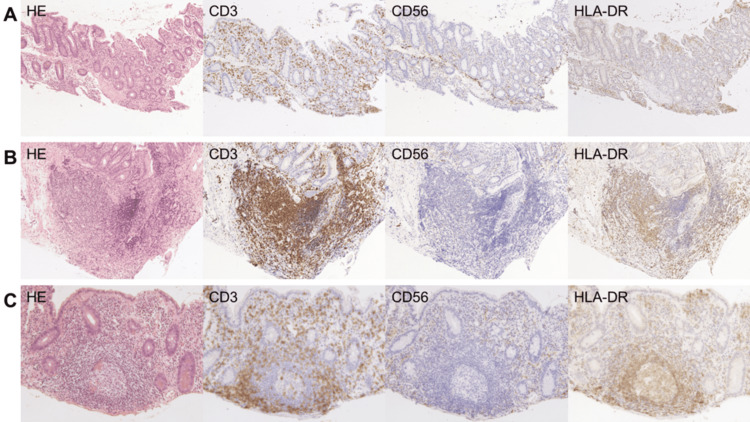
Magnified microscopic image. Magnified microscopic image of non-inflamed mucosa (A, magnification: ×10), areas with dense lymphocyte infiltration (B, magnification: ×10), and lymphoid follicles (C, magnification: ×20).

Representative histological images are shown in Figures 4-6. Histological analysis revealed that the pouch mucosa could be categorized into three distinct parts: i) non-inflamed mucosa, ii) areas with dense lymphocyte infiltration, and iii) lymphoid follicles. In the non-inflamed mucosa, CD3+ T lymphocytes were sparsely distributed, and CD56+ cells were predominantly located on the basal side of the mucosal layer (Figures 4A, 4B). HLA-DR+ cells were present in both superficial and basal regions of the mucosal layer. In areas with dense lymphocyte infiltration, CD20+ B and CD3+ T lymphocytes were intermingled, whereas CD56+ cells were scarcely observed (Figures 5A, 5B, white arrows). HLA-DR+ cells were relatively concentrated in these areas, partially overlapping the localization of CD3+ T lymphocytes. In contrast, within the lymphoid follicles, CD20+ B and CD3+ T lymphocytes were spatially segregated (Figures 4A, 5B, black arrows). CD20+ B lymphocytes were centrally clustered in a circular pattern surrounded by CD3+ T lymphocytes. CD56+ cells are rarely present in lymphoid follicles, whereas HLA-DR+ cells are found in the same locations as CD20+ B lymphocytes.

**Figure 7:**
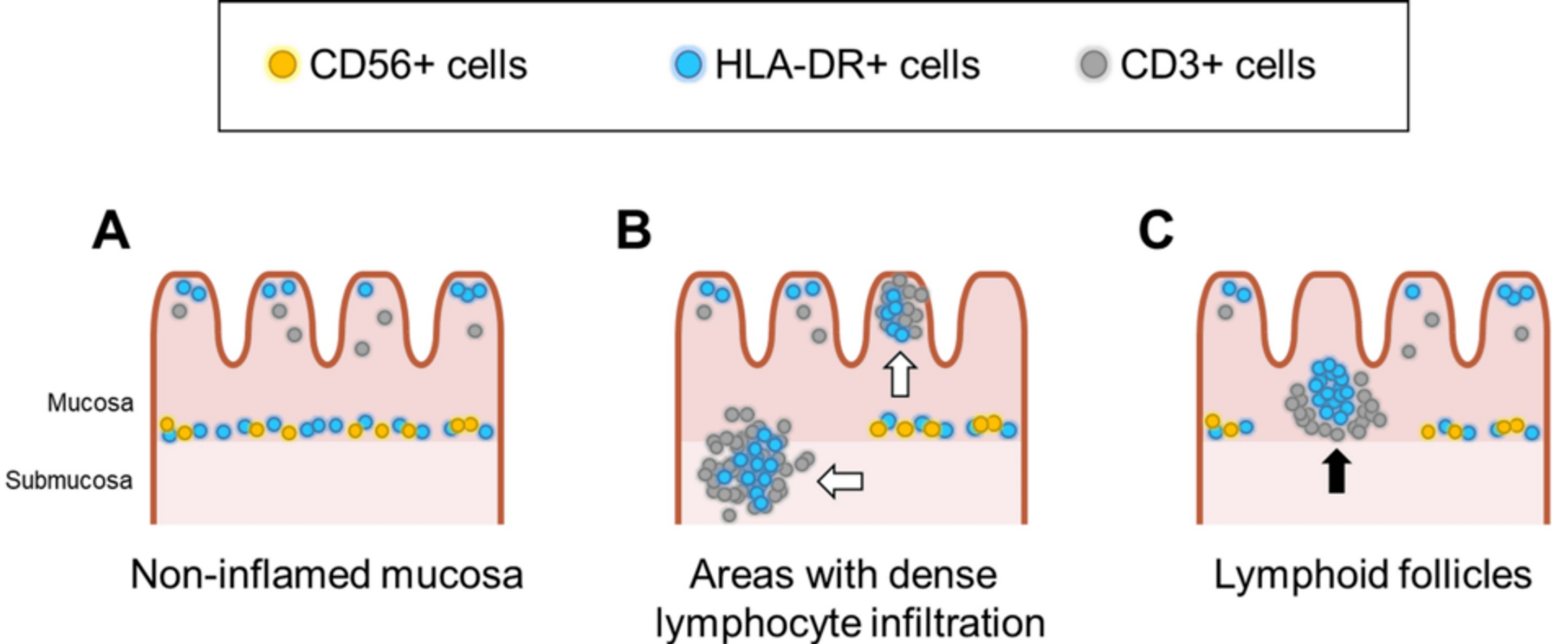
Schematic illustrations of three distinct areas: non-inflamed mucosa (A), areas with dense lymphocyte infiltration (B), and lymphoid follicles (C). Image credits: Iwamuro M, Tanaka T, Takahara M, Inokuchi T, Hiraoka S.

Figures 7A-7C show a schematic illustration of the localization of CD56+, HLA-DR+, and CD3+ cells across three distinct regions: non-inflamed mucosa, areas with dense lymphocyte infiltration, and lymphoid follicles.

## Discussion

In the present study, flow cytometry analysis revealed that the CD56+/CD3+ and CD8+/CD3+ ratios were lower in the UC-UE group than in the FAP group. These findings are consistent with those of our previous study in patients with ulcerative colitis [8]. In that study, we observed that the CD56+/CD3+ and CD8+/CD3+ ratios were lower in the rectum of ulcerative colitis patients with inflammation (Mayo endoscopic sub-score 1 or 2) than in those without inflammation (Mayo endoscopic sub-score 0). In ulcerative colitis, a Th2-type immune response predominates, leading to an increase in CD4+ T cells and the release of cytokines that promote inflammation [9-12]. Therefore, despite the lack of a difference in the CD4+/CD3+ fraction, the decrease in the CD8+/CD3+ ratio in pouchitis patients with ulcerative colitis is consistent with previous reports. CD56+CD3+ cells, also known as natural killer T (NKT) cells, play crucial roles in immune regulation. A reduction in NKT cells is associated with an increased risk of autoimmune diseases and chronic inflammation [13-16]. Research indicates that NKT cells are involved in protecting against autoimmune conditions such as type 1 diabetes, experimental autoimmune encephalomyelitis, and inflammatory bowel diseases. Recent studies have also highlighted that CD56+ NKT cells tend to decrease in certain pathological conditions [17,18]. Moreover, these cells play a crucial role in immune surveillance, particularly in cancer and autoimmune diseases, and are strongly linked to cytotoxic responses. Therefore, a decrease in the number of NKT cells may contribute to the onset or exacerbation of pouchitis.

Although variations were observed in the CD56+/CD3+ and CD8+/CD3+ ratios, the lack of differences in the other analyzed parameters was unexpected. This study was limited by a relatively small sample size of 15 patients, which likely influenced the results. However, in our previous study of 18 patients who did not undergo ileal pouch-anal anastomosis surgery (13 patients with ulcerative colitis and five patients with non-inflammatory bowel disease), we observed distinctions in a greater number of parameters [8]. We performed an immunostaining analysis to elucidate why differences in the lymphocyte fraction composition were rarely observed. As shown in the schematic illustrations in Figure 6, immunostaining revealed that CD56+, HLA-DR+, and CD3+ cells exhibited distinct distribution patterns among the following three segments: i) non-inflamed mucosa, ii) areas with dense lymphocyte infiltration, and iii) lymphoid follicles. Although flow cytometric analysis did not reveal significant differences in the HLA-DR+/CD3+ subset among the groups, immunostaining revealed a higher abundance of HLA-DR+ cells in the infiltrating lymphocyte population. HLA-DR+ activated T cells, a marker of immune activation, are frequently elevated in conditions such as autoimmune diseases and inflammatory responses [17,19]. Therefore, the increase in HLA-DR+ T cells observed in the infiltrating lymphocyte population in our study likely indicates heightened immune activation, possibly driven by chronic inflammation or infection. We believe that both of these changes-the decrease in CD56+ NKT cells and the increase in HLA-DR+ activated T cells-are consistent with immune dysregulation commonly observed in various disease models.

We speculate that the heterogeneity and uneven distribution of lymphocyte composition in the pouch mucosa may explain the difficulty in detecting differences using flow cytometry. Areas with dense lymphocyte infiltration were not uniformly observed throughout the biopsy specimens. Still, they were present in a patchy distribution (Figure 5A, white arrows), with portions of non-inflamed mucosa also present. In some cases, lymphoid follicles were intermixed (Figure 5B, black arrows). Moreover, lymphoid follicles were observed even in cases with an intact mucosa (Figure 4A). Given that the ileum naturally contains small clusters of lymphatic tissue known as Peyer’s patches [20-23], the observation of lymphoid follicles in the non-inflamed ileal pouch mucosa is expected.

Landy et al. conducted a pouch mucosa flow cytometry analysis, focusing on dendritic cells [24]. Although an increased proportion of dendritic cells expressing Toll-like receptors is generally observed in inflamed tissues from patients with ulcerative colitis who have not undergone ileal pouch-anal anastomosis, there were no significant differences in Toll-like receptor expression between the non-inflamed pouches of patients with ulcerative colitis and those with FAP. Considering previous research findings and our results regarding HLA-DR+ cells, the accurate analysis of immune cells in pouchitis using flow cytometry presents significant challenges, as isolated lymphocytes have lost their positional context. Techniques such as multiplex staining, multiplexed ion beam imaging, and spatial gene expression analysis are required to analyze the spatial distribution of lymphocytes and simultaneously characterize these cells in detail. Employing these methods will deepen our understanding of the pathophysiology of pouchitis.

This study has several limitations. First, the relatively small sample size of 15 patients may restrict the generalizability of the findings. A larger cohort would be beneficial for validating the observed differences in lymphocyte populations. Second, variability in the timing of sample collection post-surgery could introduce inconsistencies in lymphocyte profiles, highlighting the need for more uniform timing to minimize this variability. Lastly, the study's cross-sectional nature limits the ability to observe changes in lymphocyte profiles over time or in response to treatment. Longitudinal studies are needed to provide deeper insights into the progression of pouchitis and the evolving role of lymphocyte populations.

## Conclusions

This study revealed a reduction in NKT cells and an increase in HLA-DR+ cells in the inflamed areas of the ileal pouch of patients with ulcerative colitis. Although flow cytometric analysis did not show significant differences in HLA-DR+ cells, immunostaining revealed the spatial distribution of lymphocytes, highlighting the distinctions between non-inflamed mucosa, areas of dense lymphocyte infiltration, and lymphoid follicles. These findings highlight the need to focus on these three components in future studies to understand the immunological mechanisms underlying pouchitis better.
